# Food Consumption According to the NOVA Food Classification and Its Relationship with Symptoms of Depression, Anxiety, and Stress in Women

**DOI:** 10.3390/nu16213734

**Published:** 2024-10-31

**Authors:** Daniel Emiliano de Farias Xavier, Rúbia Cartaxo Squizato de Moraes, Thallyta Alanna Ferreira Viana, Joicy Karla Grangeiro Pereira, Paulo César Trindade da Costa, Davyson Barbosa Duarte, Melyssa Kellyane Cavalcanti Galdino, Adélia da Costa Pereira de Arruda Neta, José Luiz de Brito Alves, Vinícius José Baccin Martins

**Affiliations:** 1 Department of Nutrition, Federal University of Paraíba (UFPB), João Pessoa 58051-900, Brazil; daniiel428@gmail.com (D.E.d.F.X.); rubiacartaxo@gmail.com (R.C.S.d.M.); thallytaviana@hotmail.com (T.A.F.V.); joicykarla21@hotmail.com (J.K.G.P.); pctc@academico.ufpb.br (P.C.T.d.C.); jose.luiz@academico.ufpb.br (J.L.d.B.A.); 2 Laboratory of Nutrition, Physical Activity and Phenotypic Plasticity, Academic Center of Vitória, Federal University of Pernambuco (UFPE), Vitória de Santo Antão 55608-680, Brazil; davyson.duarte@ufpe.br; 3 Department of Psychology, Federal University of Paraíba (UFPB), João Pessoa 58051-900, Brazil; melyssa.cavalcanti@academico.ufpb.br; 4 Center for Food Studies and Research, State University of Campinas (UNICAMP), Campinas 13083-590, Brazil; adeliacpereira@gmail.com; 5 Department of Physiology and Pathology, Federal University of Paraíba (UFPB), João Pessoa 58051-900, Brazil

**Keywords:** anxiety, depression, food consumption, NOVA classification, stress, women

## Abstract

Background/Objectives: Depression, anxiety, and stress are highly prevalent mental disorders worldwide, and food consumption can change in individuals with these conditions. We aimed to assess the food consumption of women with depressive symptoms and compare it to a control without symptoms. Methods: A cross-sectional study was conducted with 96 women, aged 18–59, allocated into two groups: control *(n* = 62) or depressive symptoms (*n* = 34). The participants underwent an anthropometric assessment, and food consumption was evaluated using a 24 h food recall and the NOVA classification. Depressive symptoms, anxiety, and stress were measured using the DASS-21 questionnaire. Results: Anthropometric parameters did not differ between the groups. Women with depressive symptoms consumed fewer calories (*p* = 0.006), carbohydrates (*p* = 0.014), proteins (*p* = 0.036), and lipids (0.011) from unprocessed and minimally processed foods (UMPF) compared to the control women. A negative correlation was found between the dietary consumption energy of UMPF and symptoms of depression (r = −0.337; *p* = 0.001), anxiety (r = −0.262; *p* = 0.014), and stress (r = −0.260; *p* = 0.014), as well as a positive correlation between energy intake from ultra-processed foods (UPF) and symptoms of depression (r = 0.218; *p* = 0.042) and stress (r = 0.227; *p* = 0.034). Regression analysis showed that depressive symptoms accounted for 7.6% of the lower energy consumption from UMPF. Conclusions: Women with depressive symptoms displayed lower UMPF consumption, and this was negatively correlated with symptoms of depression, anxiety, and stress. Professional dietary advice can improve health status in these patients.

## 1. Introduction

Mental health disorders, including depression, can significantly impact quality of life, personal relationships, and daily productivity [1]. Depression has a multifactorial etiology, with individual, familiar, and systemic (economic, justice, social, and environmental) factors leading to the disorder. It is estimated that more than 280 million people worldwide suffer from depressive disorders, with women being the most affected [2]. In Brazil, 10.2% of people over the age of 18 report being diagnosed with depression, equivalent to approximately 16.3 million people [3].

Although unhealthy eating is widely associated with the development of non-communicable diseases, the consumption of ultra-processed foods has also been linked to the development of depressive symptoms in the USA [4], Korea [5], Italy [6], and Brazil [7].

In a study aimed at evaluating the relationship between ultra-processed food consumption, depression, and brain volume in adults, participants with higher consumption of ultra-processed foods were found to have a smaller volume in the limbic system. However, these associations were partially influenced by obesity [8]. Young Italian adults were stratified into quartiles based on ultra-processed food consumption. While no significant differences were observed in carbohydrate and protein intake between the groups, the amount of fat was significantly higher in the highest quartile. In the highest quartile, ultra-processed food consumption was associated with at least an 89% increased risk of developing depressive symptoms [6].

In recent decades, consumption of processed and ultra-processed foods has increased, accounting for approximately 31% of daily calorie intake. Conversely, the consumption of unprocessed and minimally processed foods has decreased, making up 53.4% of the daily calories consumed by Brazilians [9]. Highly processed foods are typically energy-dense and high in fat, sugar, and salt, whereas foods with lower levels of processing are rich in vitamins, minerals, and fiber and are lower in calories [10]. In line with the association between the increased consumption of ultra-processed foods and the development of depressive symptoms, a systematic review found that high intake of fruits and vegetables is linked to beneficial effects on mental health [11]. The authors proposed two possible pathways: one being the direct impact of nutrients found in natural foods in the brain, such as vitamins C and B, carotenoids, and polyphenols, and the other suggesting that individuals with better mental health are more likely to maintain a healthier diet.

Depressive symptoms are not the only consequence of the increased consumption of ultra-processed foods. This dietary pattern is also associated with the development of anxiety (affecting 300 million people in the world [2]) and stress, and the relationship appears to be bidirectional [12]. Individuals undergoing pharmacological treatment for mental health disorders such as depression may experience changes in food intake and weight, either because of the depression itself or due to the effects of medication on brain centers regulating food intake. In this context, we hypothesize that women with depressive symptoms in a non-clinical sample exhibit altered food intake, characterized by the increased consumption of palatable foods and/or a decrease in UMPF. Our primary objective was to evaluate the food consumption of women with symptoms of depression and compare them with those of the control women. The secondary objective was to assess the relationship between food consumption, anxiety, and stress.

## 2. Methods

### 2.1. Participants and Study Design

This was cross-sectional study involving women aged 18 to 59 years, recruited from six municipal schools in João Pessoa as part of a larger study conducted between March 2021 and June 2023, focusing on children and their mothers. The women recruited for the study were in good health, with no diagnosed psychiatric illnesses, and were not undergoing any treatment for mental health conditions. Group stratification was based on depressive symptoms obtained using Depression, Anxiety, and Stress Scale (DASS-21). According to this scale, the women were classified into five groups of symptoms of depression: normal, mild, moderate, severe, and very severe. For analysis, they were allocated into two groups: control group (n = 62), without any symptoms of depression, and depressive symptoms group (n = 34) for any level of depressive symptoms (mild/moderate/severe/very severe).

A total of 128 women were invited to participate in this study; however, 32 either declined to participate or were excluded due to missing health data, incomplete questionnaires, or non-participation in 24 h dietary recalls. The exclusion criteria were diagnosed psychiatric diseases, cancer, and pregnancy; however, no women were excluded based on these criteria.

This study was conducted in accordance with Declaration of Helsinki and was approved by the Research Ethics Committee of the Health Sciences Center of the Federal University of Paraíba—UFPB, under CAAE: 53905321.9.0000.5188. All women who agreed to participate provided signed consent before start the study.

### 2.2. Dietary Data and Food Consumption

The 24 h recall method quantifies all food and beverage consumption from the previous day. Administering it on both weekdays and weekends, as was done in this study, enhances the accuracy in estimating overall food intake [13]. During interviews conducted by trained nutritionists, participants used a Global Diet photo album to help estimate portion sizes. Women reported all foods and beverages consumed in the previous 24 h, including details such as preparation methods, brands of processed and ultraprocessed foods, and portion sizes.

These recalls provided calorie and nutrient intake information, and foods were classified according to the NOVA classification system [14]. The NOVA classification categorizes foods based on their degree of processing into four groups: unprocessed or minimally processed foods, culinary ingredients, processed foods, and ultra-processed foods. The foods collected were categorized as follows: (1) UMPF, foods consumed in their natural state or with minimal processing such as fruit, vegetables, grains, fresh meat, fish, eggs, milk, roots, and tubers; (2) processed foods, foods that have substances added to increase shelf life or improve flavor, including bread, cheeses, yogurts, cakes, and pasta; (3) UPF, foods that undergo several stages of processing and contain preservatives, colorings, stabilizers, and/or artificial flavorings such as soft drinks, filled cookies, snacks, instant noodles, and processed meat.

In this study, foods reported in the 24 h recalls were classified into these groups, and the multiple source method (MSM) was used to calculate the calories and grams of carbohydrates, proteins, and lipids for each processing category. Data analysis was performed using Brasil Nutri, Stata software (v.15), and MSM. For this study, we used three categories out of four, namely unprocessed or minimally processed, processed, and ultra-processed.

### 2.3. Measuring Symptoms of Depression, Anxiety, and Stress

The assessment was conducted using the Depression, Anxiety, and Stress Scale (DASS-21) questionnaire, which consists of 21 items: 7 items for assessing depression, 7 for anxiety, and 7 for stress. Participants answered each item based on their experiences over the past week, using a 4-point Likert scale ranging from 0 (not at all applicable to me) to 3 (very applicable to me). Scores are interpreted as follows: very severe > 19; severe 19–15; moderate 14–10; mild 9–8; no symptoms/normal 7–0. The questionnaire was assessed and interpreted by trained psychologist. The DASS-21 has adequate psychometric properties, including validity, reliability, sensitivity, and specificity, and has been translated and validated for the Brazilian population, with Cronbach’s alpha of 0.92 for depression, 0.90 for stress, and 0.86 for anxiety [15].

### 2.4. Socioeconomic and Anthropometric Data

Socioeconomic status was assessed using a questionnaire on monthly income.

Weight was measured with a calibrated digital scale (Omron–HBF–514, Kyoto, Japan), with the participants positioned in the center of the scale wearing minimal clothing. Height was measured using a portable stadiometer (Alturaexata, Belo Horizonte, Brazil), with the participant standing upright. Body mass index (BMI) was calculated by dividing weight (kg) by height (m) squared. Waist circumference and hip circumferences were assessed with a measuring tape. All measurements were taken in triplicate, and then the–arithmetic mean was calculated. Triceps, subscapular, and thigh skinfolds were assessed using a clinical adipometer (Sanny, São Bernardo do Campo, Brazil), and these measurements were used to calculate body fat and fat-free mass according to Jackson [16]

### 2.5. Protocol

The women who met the eligibility criteria were invited to participate in the study. After agreeing and signing the informed consent form, they completed a socioeconomic questionnaire. On the second day of the study, they underwent an anthropometric assessment, completed the DASS-21 questionnaire, and filled out the first 24 h dietary recall, which referred to a weekday. Participants were allocated to their respective groups only after completing the DASS-21 questionnaire. On the third day, which was a Monday, the second 24 h dietary recall, referring to a weekend day, was administered.

### 2.6. Statistical Analysis and Sample Size

The normality of the variables was assessed using the Shapiro–Wilk test. Categorical data were analyzed using the chi-square test. For comparison between groups, Student’s *t*-test or Mann–Whitney test was applied. Results were expressed as means and standard deviations or *n* (%) for these analyses. Multiple regression and partial correlation analyses were adjusted for BMI. Statistical significance was defined as alpha < 0.05. Statistical analysis was performed using the Statistical Package for Social Sciences (SPSS) software, version 21.0 (IBM Inc., Chicago, IL, USA). Participants with missing data were handled by excluding only the affected variables, not the entire individual, except in regression analysis, in accordance with SPSS default.

A posteriori power calculation was performed using GPower 3.1.9.7 (University of Kiel, Kiel, Germany) to verify the power of Student’s *t*-test and multiple regression. The calculation indicated that a sample size of 96 subjects (62 in the control group and 34 in the group with symptoms of depression), with an effect size of 0.5 and an alpha of 0.05, would provide a power of 64% for Student’s *t*-test and 85% for multiple regression.

## 3. Results

Table 1 shows the anthropometric and body composition characteristics, anxiety and stress level and income of the groups. Age, weight, BMI, waist circumference, hip circumferences, body composition and income did not differ between the groups. However, anxiety and stress levels were significantly higher in the group with depressive symptoms compared to the control group.

The consumption of calories, proteins, carbohydrates, and lipids according to the NOVA food classification is shown in Table 2. Energy and consumption of macronutrients originating from UMPF were significantly lower in the depressive symptoms group compared to the control group. No difference was found in the consumption of calories, proteins, carbohydrates, and lipids from processed food and UPF between the groups.

Table 3 shows the partial correlation between mental health and food consumption according to the NOVA classification, adjusted for BMI. There was a negative correlation between symptoms of depression, anxiety, stress, and total DASS-21 score with energy, protein, and lipids in the UMPF groups. Additionally, depressive symptoms were negatively correlated with carbohydrate intake. No significant correlation was found between mental health and the consumption of processed food. However, there was a positive correlation between depressive symptoms and both energy and lipid intake from UPF and between stress and total score of DASS-21 with energy and carbohydrate intake derived from UPF.

Multiple regression analysis predicting energy consumption according to the NOVA classification, adjusted for BMI, is shown in Table 4. Symptoms of depression, anxiety, and stress did not explain the consumption of energy from processed food and UPF. However, only depression symptoms accounted for 7.6% of the lower consumption of UMPF.

## 4. Discussion

In this study, food consumption categorized by the NOVA classification was analyzed in women without and with depressive symptoms. It was found that women with depressive symptoms consumed fewer minimally processed foods compared to the control group. In addition, symptoms of depression, anxiety, and stress showed an inverse correlation with the consumption of macronutrients derived from unprocessed and minimally processed food groups. However, no differences in anthropometric parameters were found between the groups.

Many studies compare the consumption of ultra-processed foods using quartiles or tertiles of consumption, with findings suggesting that the highest levels are associated with increased odds of developing depression [17]. Contreras-Rodrigues et al. [8] compared non-obese participants (control) with obese individuals and found no significant differences in energy intake, fatty acids, or carbohydrates but observed a higher intake of ultra-processed foods in the group with obesity. In the present study, we compared the consumption of ultra-processed foods between participants with and without depressive symptoms and the consumption of UPF did not differ between groups, although positive correlations were found between symptoms of depression and stress with energy from UPF, as well as between depression and lipids, and carbohydrates with stress adjusting for BMI. Multiple regression analysis indicates that symptoms of depression, but not anxiety or stress, account for approximately 7.6% of the lower consumption of energy derived from UMPF, after adjusting for BMI. In this sense, the findings suggest that depressive symptoms may be associated with a higher energy intake derived preferentially from ultra-processed foods, which are highly palatable, leading to a reduction in the consumption of unprocessed and minimally processed foods (UMPF).

It has been found that diets rich in fruits, vegetables, nuts, olive oil, poultry, fish, and unprocessed meat were negatively associated with the risk of depression, while sweetened beverages, fried foods, snacks, pastries, and processed meat were positively associated with an increased risk of depression [18]. In the present study, the UMPF included fruits, greens and vegetables, and red meat. The impact of red meat consumption on depression remains unclear. In a cross-sectional study that aimed to analyze the consumption of red meat and its relationship with depression, anxiety, and stress using the DASS-21 scale, women were classified into quartiles of red meat consumption [19]. The authors found that those in the highest quartile had a higher prevalence of depressive symptoms, anxiety, and stress compared to those in the lowest quartile. In a study involving 435 women, the relationship between diets containing less processed foods and symptoms of depression, anxiety, and stress was also evaluated using the DASS-21 scale. Women who followed Mediterranean or Paleolithic dietary patterns, with low consumption of red and processed meat, had a lower risk of psychological disorders such as depression, anxiety, and stress. These benefits were attributed to the antioxidants, fiber, and micronutrients in these diets, which can improve inflammation, decrease oxidative stress, and balance the gut microbiome, all factors crucial for mental health [20].

On the other hand, a scoping review [21] did not find differences between meat consumers and non-consumers regarding positive psychological variables, such as life satisfaction, positive mental health, optimism, positive emotions, and psychological well-being. Consistent with our findings, Coletro et al. [22] found that adult men and women, in a sample with a 15.6% prevalence of depression, with higher consumption of fresh and minimally processed foods, including beans, vegetables, fruits, eggs, chicken, fish, and red meat, had a lower prevalence of symptoms of depression. It is important to highlight that in the present study, red meat was not studied in isolation, and other components of the diet, such as fruits and vegetables, may have contributed to the lower level of depressive symptoms.

Similar to depressive symptoms, the consumption of ultra-processed foods has been associated with anxiety, while vegetables and fruits have been linked to lower levels of anxiety [23]. In a cross-sectional study, where patients were allocated to quintiles based on ultra-processed food consumption, those in the highest quintile were more likely to have anxiety disorders [24]. Coletro et al. [22] also found a higher consumption of ultra-processed foods, in a sample with 23.3% of participants diagnosed with anxiety. Although in the present study, anxiety did not impact the consumption of UMPF, processed or ultra-processed in multiple regression analysis, it was found to be negatively correlated with energy, protein, and lipids from UMPF adjusted for BMI. The components of a natural diet are rich in essential micronutrients such as B vitamins, vitamin D, and zinc, which contribute to brain health and may help reduce the prevalence of depression and anxiety [25].

In a systematic review aimed at evaluating the association between junk food and mental health, it was found that junk food consumption increases the odds of developing stress [26]. Conversely, stress can alter food choices, often leading to increased calorie intake, particularly from palatable foods that are high in sugar, fat, and calories [27]. This relationship explains, at least in part, the negative correlation found between stress and energy intake from UMPF, as well as the positive correlation with UPF in the present study.

Although anxiety and stress did not predict the higher consumption of ultra-processed foods or lower consumption of minimally processed foods, the present study found a decrease in UMPF consumption in relation to patients with depressive symptoms. Anxiety and stress are physiological responses that, at adequate levels, can be part of normal life. While individuals may occasionally feel depressed, the DASS questionnaire specifically assesses depressive symptoms. Therefore, the levels of stress and anxiety identified in this study may not have been high enough to induce significant changes in eating behavior. Our sample of participants was not recruited from clinical settings for the treatment of physical or mental illnesses. In this sense, the participants in this study did not have a medical diagnosis of depression, despite having higher DASS-21 scores, as this scale cannot be used for diagnosing depression. However, they did exhibit depressive symptoms; that is, although they did not have the disorder, they showed symptoms indicative of this condition.

Over the last 30 years, the cost of minimally processed foods or culinary ingredients in Brazil has increased, while the prices of UPF have decreased, making them more affordable than UMPF [28]. The financial cost of accessing natural foods can be a limiting factor, especially for poorer populations. In unemployed women with symptoms of depression, the lower consumption of UMPF was associated with the higher cost of these foods compared to UPF [29]. This suggests that socioeconomic status affects the ability to purchase UMPF, as these products tend to be more expensive than UPF. Additionally, this study shows that symptoms of depression in women are associated with the lower consumption of UMPF, especially fruit, and this relationship was mediated by income. In the present study, no difference was found in monthly income between the groups. In this context, the lower consumption of UMPF in individuals with depressive symptoms may not be mediated by income but rather influenced by food choices.

A limitation of this study is the relatively low number of participants with depressive symptoms. It is important to note that these women were not recruited from referral centers for treatment of mental health disorders, but rather from a random community sample, just like the control group participants. Another limitation is the varying severity of depression within the group, ranging from mild to very severe, as classified by the DASS-21 questionnaire. We did not use the food-frequency questionnaire, which explores food consumption over a longer period than the 24 h recall. The use of the 24 h recall method has been documented in several studies, as presented in a systematic review [12]. Furthermore, in addition to the recall, DASS-21 assesses symptoms over the previous week, thereby representing short-term conditions. The level of physical activity can have an impact on anthropometric parameters, but in our study, this variable was not assessed.

## 5. Conclusions

The present study found that women with depressive symptoms had a lower intake of UMPF, and this type of food was negatively correlated with symptoms of depression, anxiety, and stress. Although no increase in the consumption of processed or ultra-processed foods was found in women with depressive symptoms, a positive correlation was found between energy from UPF and both depression and stress. Given the limited sample size, these results should be interpreted with caution. Moreover, as the study is cross-sectional, no causal relationships can be established.

It is important to emphasize that this study is observational, and the intake of natural foods should not be viewed as a standalone solution for the treatment of depression, anxiety, or stress. These conditions require treatment from a multidisciplinary team, including physicians, psychologists, and nutritionists. Dietary and nutritional recommendations for individuals with these symptoms should be based on professional consultation, tailored to the specific needs of each patient.

## Figures and Tables

**Table 1:** Anthropometric parameters, body composition, anxiety and stress level, and income among the studied groups.

Variables	Control (*n* = 62)	Depressive Symptoms (*n* = 34)	*p*-Value
Age (years)	39.21 ± 8.28	37.38 ± 7.28	0.281
Weight (kg)	73.85 ± 16.12	76.88 ± 15.47	0.367
BMI (kg/m^2^)	29.26 ± 6.06	29.56 ± 5.68	0.808
WC (cm)	88.27 ± 12.58	89.78 ± 11.49	0.557
HC (cm)	107.74 ± 12.35	110.10 ± 12.51	0.371
Fat (kg)	23.31 ± 8.72	24.14 ± 11.22	0.697
FFM (kg)	50.14 ± 8.54	51.93 ± 6.62	0.303
Anxiety *	2.00 (0.00–6.00)	18.00 (12.00–23.00)	<0.001
Stress *	6.00 (2.00–10.00)	22.00 (14.00–34.00)	<0.001
Income up to one MW (*n* (%))	35 (37.2)	20 (21.3)	0.963
Income greater than one MW (*n* (%))	25 (26.6)	14 (14.9)	
Normal weight (*n* (%))	15 (24.2)	6 (17.6)	0.458
Weight excess (*n* (%))	47 (75.8)	28 (82.4)	

Student’s *t*-test was applied, and data are expressed as mean and standard deviation. * Mann–Whitney test was applied to anxiety and stress levels, and data are expressed as median and interquartile range. Income and nutritional status were analyzed using the chi-square test, and the values are expressed as *n* (%). BMI, body mass index; WC, waist circumference; HC, hip circumference; FFM, fat-free mass; MW, minimum wage (USD 264.37).

**Table 2:** Analysis of food consumption according to the NOVA classification between the groups.

Variables	Control (*n* = 62)	Depressive Symptoms (*n* = 34)	*p*-Value
Total energy (Kcal)	1655.50 ± 392.31	1675.42 ± 452.45	0.822
Unprocessed and minimally processed foods			
Calories (Kcal)	871.80 ± 161.61	765.36 ± 201.09	0.006
Proteins (g)	53.76 ± 9.29	48.38 ± 11.39	0.014
Carbohydrates (g)	102.53 ± 29.22	89.31 ± 29.03	0.036
Lipids (g)	29.58 ± 4.22	27.09 ± 5.00	0.011
Processed foods			
Calories (Kcal) *	414.86 (290.47–514.95)	440.52 (336.17–586.20)	0.226
Proteins (g) *	18.28 (12.42–22.94)	21.30 (12.36–28.80)	0.154
Carbohydrates (g) *	49.20 (31.22–65.44)	53.45 (38.19–65.52)	0.366
Lipids (g) *	15.74 (11.12–20.68)	17.30 (12.19–26.29)	0.118
Ultra-processed foods			
Calories (Kcal) *	311.83 (214.46–452.73)	379.09 (246.36–571.69)	0.083
Proteins (g) *	6.88 (4.13–12.61)	10.10 (6.30–14.34)	0.080
Carbohydrates (g) *	47.95 (36.45–61.95)	57.35 (41.26–76.64)	0.056
Lipids (g) *	9.98 (5.29–16.64)	13.28 (7.65–19.07)	0.076

* Mann–Whitney test and data are expressed as median and interquartile range. Student’s *t*-test. Data are expressed as mean and standard deviation.

**Table 3:** Partial correlation adjusted by the body mass index between mental health variables and food consumption according to NOVA classification.

Variables	Depression	Anxiety	Stress	Total Score †
Energy UMPF	−0.337 **	−0.262 *	−0.260 *	−0.306 **
Protein UMPF	−0.328 **	−0.263 *	−0.256 *	−0.302 **
Carbohydrate UMPF	−0.226 *	−0.128	−0.178	−0.190
Lipids UMPF	−0.313 **	−0.270 *	−0.236 *	−0.292 **
Energy PF	0.061	0.089	0.068	0.078
Protein PF	0.074	0.092	0.087	0.091
Carbohydrate PF	0.044	0.000	−0.023	0.006
Lipids PF	0.083	0.150	0.157	0.141
Energy UPF	0.218 *	0.180	0.227 *	0.224 *
Protein UPF	0.120	0.107	0.182	0.149
Carbohydrate UPF	0.208	0.176	0.245 *	0.227 *
Lipids UPF	0.228 *	0.142	0.181	0.197

UMPF, unprocessed and minimally processed foods; PF, processed foods; UPF, ultra-processed foods. † Total score of DASS questionnaire. * *p* < 0.05. ** *p* < 0.01.

**Table 4:** Multiple regression analysis predicting energy according to NOVA classification and symptoms of depression, anxiety, and stress adjusted by body mass index.

Variables	B	Beta Coefficient	*p*-Value	*p*-Value (ANOVA)	Adjusted R Square
Unprocessed and minimally processed foods					
Constant	941.425		<0.001	0.031	0.076
BMI	−2.237	−0.073	0.491		
Depression	−120.843	−0.312	0.031		
Anxiety	46.032	0.123	0.440		
Stress	−56.336	−0.144	0.326		
Processed foods					
Constant	402.556		<0.001	0.846	0.001
BMI	0.054	0.002	0.987		
Depression	16.260	0.044	0.768		
Anxiety	35.428	0.100	0.551		
Stress	−3.366	−0.009	0.953		
Ultra-processed foods					
Constant	361.297		0.008	0.367	0.004
BMI	0.022	0.001	0.996		
Depression	120.063	0.230	0.123		
Anxiety	41.909	0.083	0.614		
Stress	−84.834	−0.161	0.291		

Presence of symptoms of depression, anxiety, or stress: (0 without and 1 with symptoms).

## Data Availability

The data that support the findings of this study are available from the corresponding author upon reasonable request.
